# Understanding the Utility of Fecal Occult Blood Testing in Hospitalized Patients With Suspected GI Bleeding

**DOI:** 10.7759/cureus.57406

**Published:** 2024-04-01

**Authors:** Priyam Doshi, Corey Sievers

**Affiliations:** 1 Internal Medicine, Western Reserve Hospital, Cuyahoga Falls, USA; 2 Gastroenterology, Western Reserve Hospital, Cuyahoga Falls, USA

**Keywords:** colorectal cancer, gastrointestinal bleed, value in health care, screening tool, inpatient fobt

## Abstract

Colorectal cancer (CRC) is one of the leading causes of cancer-related mortality worldwide. There have been increasing efforts to reduce its incidence and mortality. Screening plays a crucial role, with various tests such as the fecal occult blood test (FOBT), colonoscopy, and flexible sigmoidoscopy commonly used for investigation. FOBT is a Food and Drug Administration (FDA) approved screening tool commonly used in acute healthcare settings for early detection of CRC.

We report a 50-year-old man presenting with shortness of breath, chills, and malaise with findings positive for pneumonia. Laboratory tests revealed anemia as an incidental finding. A subsequent FOBT came back positive, and the patient was admitted for further gastrointestinal testing. Esophagogastroduodenoscopy (EGD) and colonoscopy were performed, but no significant findings were observed. This case report focuses on the overuse of FOBT testing during hospital admission, despite its limited impact on patient care in acute settings. Key takeaways include being aware of the potential for false positive and false negative results from a FOBT. Using the test carefully can help reduce both direct and indirect healthcare costs for hospitalized patients, as well as minimize the use of hospital resources. The test should primarily be used for CRC screening in the outpatient setting.

## Introduction

Colorectal cancer (CRC) is the third most common cancer-related death in the United States and has been the focus of the medical community and policymakers due to its high prevalence [1,2]. The United States Preventive Services Task Force (USPSTF) recommends that adults aged 45 to 75 undergo CRC screening [1-3]. A fecal occult blood test (FOBT) is an FDA-approved non-invasive and inexpensive stool sample test that detects non-apparent blood, possibly indicating an underlying CRC [2]. The test has shown good patient compliance [2].

Fecal occult testing has multiple types, such as guaiac-based fecal occult blood test (gFOBT), fecal immunochemical test (FIT), and FIT deoxyribonucleic acid (DNA), which are routinely used [1-4]. The gFOBT is commonly used to detect the activity of the peroxidase enzyme, which is typically found in human red blood cells [2,4]. This test is performed once a year [2,4]. On the other hand, the FIT test uses antibodies to identify blood in the stool and is also done yearly [4]. The FIT DNA test is a combination of the FIT and the detection of altered DNA in stool samples. This test is performed every three years. Additionally, flexible sigmoidoscopy, colonoscopy, and computed tomography (CT) colonography are other screening tests that are commonly used.

CRC screening is the only recommended use for FOBT, as it has been scientifically validated [1,3]. However, it has also been misused for purposes other than CRC screening [1-3]. FOBT is commonly ordered as part of a routine order set for many patients with low hemoglobin levels without identifying confounding variables. There is no evidence supporting the use of FOBT for acute hospital settings, yet it has been used for other gastrointestinal (GI) symptoms, such as abdominal pain, suspicion of GI bleeding, iron deficiency anemia, and constipation [1,2].

This case report was recently sent for the American College of Physicians' annual 2024 internal medicine meeting.

## Case presentation

**Figure 1:**
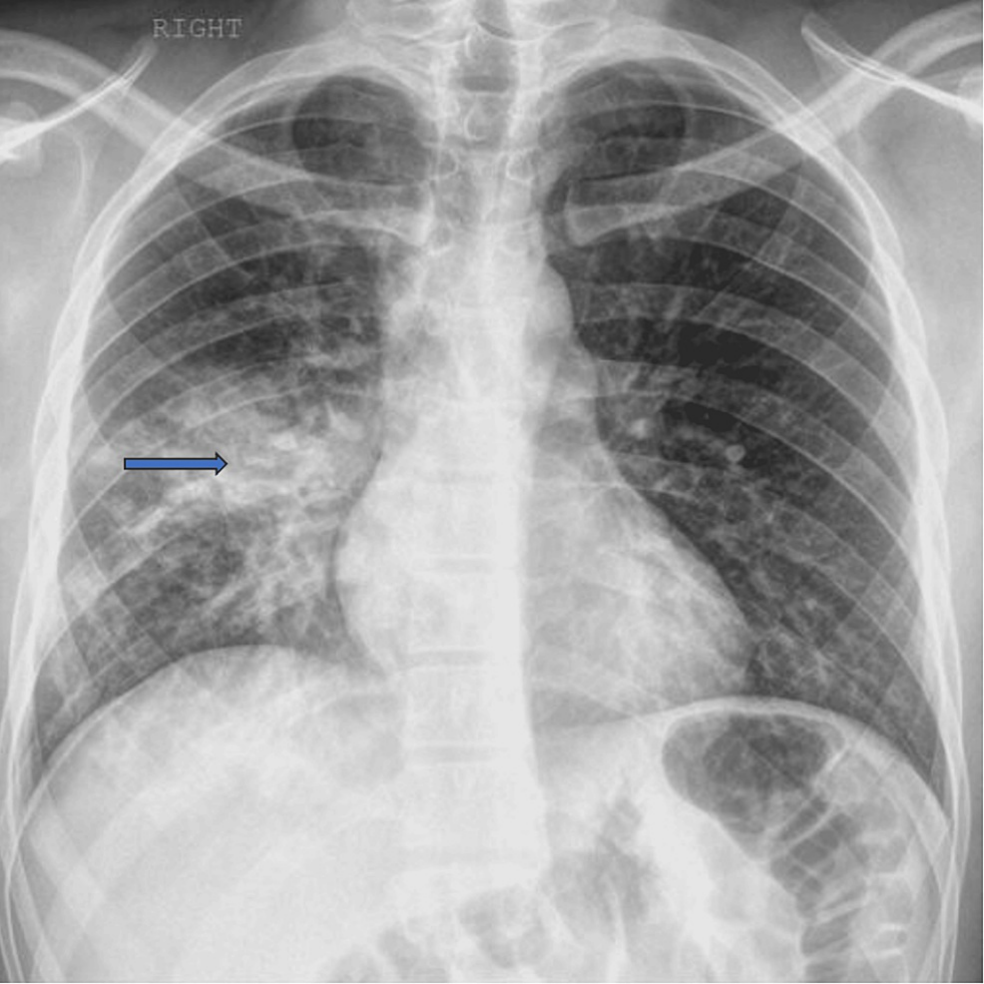
X-ray of the chest during hospital admission The blue arrow shows consolidation of the right lung lobe. The presence of other acute cardiopulmonary abnormality is ruled out.

A 50-year-old man with a history of hypertension and diabetes mellitus went to the emergency room (ER) due to difficulty breathing during physical activity. He had been feeling tired and had chills and malaise for two to three days before being admitted. Upon arrival, the patient had a normal temperature and stable vital signs while breathing room air. During the physical examination, dullness was noted on percussion, and crackles were appreciated on the right lower lung lobe. The patient was ordered a complete blood count (CBC), basic metabolic profile (BMP), and chest X-ray (CXR). Considering the patient's history and absence of GI symptoms, a stool parasite sample was not warranted. The CXR showed patchy airspace opacities on the right lobe, which confirmed the presence of pneumonia, as shown in Figure 1.

The patient was given intravenous ceftriaxone and doxycycline after sepsis was ruled out and was planned for discharge from the ER on oral antibiotics. The results of the test are shown in Table 1. The patient was new to our medical system and there were no previous labs to compare his baseline hemoglobin levels. Hence, as a part of further testing, FOBT and iron studies were also ordered. The FOBT came back positive and iron studies did not clearly point towards an iron deficiency anemia.

**Table 1:** Pertinent laboratory values on admission g/dL = gram per deciliter, % = percentage, MCV = mean corpuscular volume, FL = femtoliter, mcL = microliter, mcg/L = microgram per liter, mmol/L = millimoles per liter, mcg/dL = microgram per deciliter, ng/mL = nanogram per milliliter, TIBC = total iron-binding capacity

Laboratory Test	Result	Reference Values
Hemoglobin, g/dL	10	12-16
Hematocrit, %	34	36-48
MCV, FL	86	80-100
Platelet, mcL	200	138-367
WBC, mcL	13.5	3.6-10.3
Procalcitonin, mcg/L	0.4	<0.1
Iron, mcg/dL	51	50-170
Ferritin, ng/mL	27	8-252
TIBC, mcg/dL	451	250-450

The patient was a poor historian and had difficulty recalling any symptoms such as abdominal pain, changes in bowel habits, melena, hematochezia, family history of CRC, or unintentional weight loss. Additionally, he denied using oral anticoagulants, aspirin, or non-steroidal anti-inflammatory drugs (NSAIDs). The ER consulted the GI service, and the patient was kept NPO while receiving intravenous fluids, pantoprazole, and antibiotics. Due to anemia with no apparent cause, the patient was admitted for an in-patient stay to undergo further testing. Esophagogastroduodenoscopy (EGD) and colonoscopy showed no significant findings. Therefore, the patient had to stay for one more day using hospital resources to undergo procedures that could have been scheduled as an outpatient if the FOBT had not been tested.

No specific cause of low hemoglobin was identified during the patient's hospital stay. After ruling out major causes, and due to lack of readily available medical records, the patient was suspected to have chronically low Hb levels. In the meantime, the patient was discharged with a prescription for oral pantoprazole, cefdinir, doxycycline, and iron tablets. The patient was advised to get his blood work repeated in a few days and to attend an outpatient appointment in a week. The patient's repeat blood work showed a hemoglobin of 11.1 g/dL (gram per deciliter), and no further testing was deemed necessary. After excluding all potential causes for his positive FOBT result, the only suspected reason could be the recent use of amoxicillin to treat an ear infection. The patient is scheduled for a follow-up appointment in three months.

## Discussion

When there is suspicion of active GI bleeding, the best diagnostic tools are a thorough medical history, physical examination, trending CBC, and visual inspection of stool by the clinician.

FOBT testing has several limitations in an inpatient setting, such as noncompliance with diet restrictions and medication usage that could affect test results [5]. False positive FOBT results can occur due to inflammatory bowel disease, medications such as aspirin and NSAIDs, aspirated blood from nasopharynx or pulmonary sources, and ingestion of meat and vegetables containing peroxidase [4-6]. False negatives can result from proximal GI tract lesions and high doses of vitamin C ingestion [4]. It is not recommended to perform routine FOBT at the time of digital rectal examination as microtears during DRE could lead to false positive results [4-6].

A recent study showed that only one case of CRC was diagnosed for every 214 patients inappropriately tested with FOBT [2]. In patients with a positive FOBT and negative colonoscopy, routine endoscopy is not recommended as the number needed to scope patients to find a significant lesion is not cost-effective [4,7]. Observed melena either through rectal examination or stool has a likelihood ratio of 25 for upper GI bleeding whereas a patient's self-reporting of black, tarry stools has a likelihood ratio of 5-6 [8]. An upper GI source may further be supported by an elevated blood urea nitrogen (BUN) to creatinine ratio as blood is absorbed through the small bowel and patients have concomitant decreased renal perfusion. A BUN to creatinine ratio of more than 30 is associated with a positive likelihood ratio of 7.5 for upper GI bleeding [8].

De-adaption refers to discontinuing healthcare resources that are ineffective or inefficient in terms of their cost-benefit ratio [9]. The last study confirming the usefulness of in-hospital FOBT was conducted over 22 years ago [10]. Several studies have demonstrated that educational initiatives, such as providing a simple academic module, can reduce unnecessary FOBT usage and testing among hospitalized patients, thereby improving the appropriate utilization of healthcare resources [11]. Therefore, it is not recommended to use FOBT for evaluating GI bleeding in hospitalized patients unless further research substantiates its utility. The appropriate use of this test can help reduce direct and indirect healthcare costs for hospitalized patients by avoiding expenses associated with procedures, consultations, and fees charged by healthcare providers, as well as the use of hospital resources. This move can create a positive impact on our overburdened healthcare system. Furthermore, the test is accompanied by various confounding factors that can make its contribution to clinical decision-making almost impossible for hospitalized patients.

## Conclusions

Our article discusses the inappropriate and excessive usage of the FOBT in hospital settings. While many ERs advocate its use to evaluate anemia or iron deficiency or triage patients with bleeding, it has shown to seldom impact clinical decision-making regarding whether to refer patients for further investigation.

To achieve its primary goal of CRC screening and early detection, it is suggested to perform FOBT in the ambulatory setting, which can improve its test performance characteristics.
